# A blended eHealth intervention for insomnia following acquired brain injury: study protocol for a randomized controlled trial

**DOI:** 10.1186/s13063-020-04789-y

**Published:** 2020-10-16

**Authors:** Marthe E. Ford, Gert J. Geurtsen, Erny Groet, Coen A. M. Van Bennekom, Eus J. W. Van Someren

**Affiliations:** 1 Department of Psychology, Heliomare Rehabilitation, Wijk aan Zee, The Netherlands; 2 Department of Medical Psychology, Amsterdam UMC, location AMC, Amsterdam, The Netherlands; 3 Research and Development, Heliomare Rehabilitation, Wijk aan Zee, The Netherlands; 4 Coronel Institute for Labor and Health /Amsterdam UMC, location AMC, Amsterdam, The Netherlands; 5 Department of Sleep and Cognition, Netherlands Institute for Neuroscience, Amsterdam, The Netherlands; 6 Departments of Integrative Neurophysiology and Psychiatry, Amsterdam UMC, VU University, Amsterdam Neuroscience, Amsterdam, The Netherlands

**Keywords:** Insomnia, Sleep, Acquired brain injury, Stroke, Traumatic brain injury, Cognitive behavioral therapy, Online treatment, eHealth

## Abstract

**Background:**

Up to a third of stroke patients and patients with traumatic brain injury suffer from insomnia, including problems to fall asleep or stay asleep at night. Insomnia may exacerbate other brain damage-related problems, for example regarding cognitive functioning and emotional well-being; may lead to poorer quality of life; and may complicate recovery processes. Cognitive behavioral therapy for insomnia, delivered face-to-face or online, is found to be effective in the general population. However, despite the high prevalence and serious consequences of insomnia following acquired brain injury, studies on the efficacy of face-to-face cognitive behavioral treatment in this population are scarce, and this applies even more for studies on online cognitive behavioral therapy. Therefore, this study aims to evaluate the efficacy of a newly developed guided online cognitive behavioral therapy for insomnia following acquired brain injury.

**Methods:**

A multicenter, prospective, randomized, open-label, blinded end point study (PROBE) will be conducted, in which 48 patients diagnosed with stroke or traumatic brain injury and insomnia will be randomly allocated to the online cognitive behavioral therapy for insomnia treatment group or the treatment as usual group. The treatment consists of 6 online cognitive behavioral therapy sessions given on a weekly basis and personalized feedback after each session, combined with 2 face-to-face sessions. Outcomes will be assessed at baseline, immediately after the intervention period and at 6-week follow-up. The primary outcome is the insomnia severity assessed with the Insomnia Severity Index. Secondary outcome measures include sleep quality, sleep features derived from the sleep diary, fatigue, anxiety and depression, subjective cognitive functioning, and societal participation.

**Discussion:**

This study will provide insight on the efficacy of online cognitive behavioral therapy for insomnia following stroke and traumatic brain injury.

**Trial registration:**

Netherlands Trial Register NTR7082. Registered on 12 March 2018.

## Background

There is increasing awareness that sleep disorders following acquired brain injury are a serious problem and therefore need attention. These sleep disorders include sleep apnea, circadian rhythm disturbances, excessive daytime sleepiness, increased need for sleep, and insomnia [1–3]. The latter is most commonly reported. Complaints include trouble falling asleep, staying asleep, or waking up early and are accompanied by daytime complaints. Up to a third of stroke patients [4] and patients with traumatic brain injury (TBI) [2] meet DSM-IV criteria for insomnia disorder (ID), which is three times more than the 10% of the general population that suffers from ID [5]. Insomnia may exacerbate other brain damage-related problems, for example regarding cognitive functioning and emotional well-being; may lead to poorer quality of life; and may complicate recovery processes [2, 4, 6]. The treatment of insomnia should therefore be an important part of rehabilitation after acquired brain injury.

Numerous randomized controlled trials have shown that cognitive behavior therapy for insomnia (CBT-I) is an effective treatment for insomnia in otherwise healthy people, as well as in several populations with insomnia comorbid with another disorder [7–9]. A problem with implementation of CBT-I on a large scale is the lack of skilled therapists, limiting access to treatment. Online versions of CBT-I have been developed to reach a larger group of patients with insomnia. Recent reviews show that Internet-delivered CBT-I is effective in improving sleep in adults with insomnia [10, 11].

Despite the high prevalence and serious consequences of insomnia in stroke patients or patients with TBI, studies examining CBT-I following acquired brain injury are scarce. To date, CBT-I was examined in two studies concerning stroke patients [12, 13] and five studies concerning patients with mild to severe TBI [14–18]. The CBT-I protocol applied in the studies consisted of four to eight weekly sessions, combining cognitive and behavioral techniques including stimulus control, sleep restriction, cognitive therapy, and sleep hygiene education. CBT-I was adapted to brain injury patients by adding information on factors that could contribute to insomnia specifically after brain injury. CBT-I was administered online in one study [18] and face-to-face in the other six studies. Six out of these seven studies found significant improvement on sleep outcomes [12–14, 16–18] and four of these studies found significant improvement on secondary outcomes as depression and fatigue [12, 14, 15, 17]. Only three studies were randomized controlled trials, of which two compared CBT-I with treatment as usual [12, 14] and one compared CBT-I with a placebo condition (online education only) [18]. Furthermore, only three out of the seven studies—and none of the randomized controlled trials—formally assessed a diagnosis of ID as inclusion criterion [13, 16, 17]. More randomized controlled trials using formal diagnosis of ID are needed in order to reach a well-founded conclusion on the efficacy of CBT-I in patients with stroke or TBI.

For the present study, we developed an online eHealth CBT-I (eCBT-I) (see Table 1 for more details). Not only is eHealth easier to access and probably more cost-effective, it also has more benefits for patients, such as the opportunity to reread information and the freedom to follow treatment at their own time, place, and pace. The present study will compare eCBT-I with treatment as usual in patients with stroke or TBI to evaluate the added value on top of standard care. Treatment as usual does not address sleep.

**Table 1:** Overview of online cognitive behavioral therapy for insomnia (eCBT-I)

Week 1:	­ Face to face session to provide information about the eHealth treatment and to optimize motivation for treatment
	­ Start of online session 1: psychoeducation on sleep, the different sleep stages, and sleep disorders following acquired brain injury and their consequences in daily life. Homework assignment: map personal sleep problems and their consequences for daily life together with coping so far: what was helpful and what was not?
	­ Start with a daily sleep diary, which will be continued throughout the treatment.
Week 2:	­ Online session 2: setting personal goals for treatment, information about sleep hygiene. Homework assignment: write down sub goals to improve sleep hygiene for the following week.
Week 3:	­ Second face- to- face session to evaluate the personal goals for treatment
	­ Online session 3: information on the relation between stress and sleep and different relaxation techniques. Homework assignment: practice of these relaxation techniques the following week.
Week 4:	­ Online session 4: information on the circadian clock which is entrained by light and temperature and the influence of activation on daytime sleepiness. Homework assignment: to balance activities and relaxation or to be more active during daytime.
Week 5:	­ Online session 5: different cognitive techniques, such as mindfulness and cognitive restructuring. Homework assignment: address and change unhelpful cognitive beliefs.
Week 6:	­ Online session 6: consolidation and relapse prevention.

### Objectives

The objectives of this study are:

1. To evaluate the efficacy of eCBT-I in reducing insomnia severity posttreatment and at 6-week follow-up in patients with insomnia following a stroke or TBI, as compared to treatment as usual (TAU)
2. To evaluate the efficacy of the eCBT-I in reducing complaints about fatigue, cognitive functioning, emotional well-being, and societal participation posttreatment and at 6-week follow-up compared to TAU
3. To explore whether treatment efficacies vary with severity of insomnia, with the severity and type of brain injury, and with the time since brain injury.

Our primary hypothesis is that eCBT-I will reduce insomnia posttreatment more than treatment as usual, compared to baseline. Our secondary hypotheses are that eCBT-I will improve fatigue, cognitive functioning, emotional well-being, and societal participation.

## Methods

### Study design

**Fig. 1:**
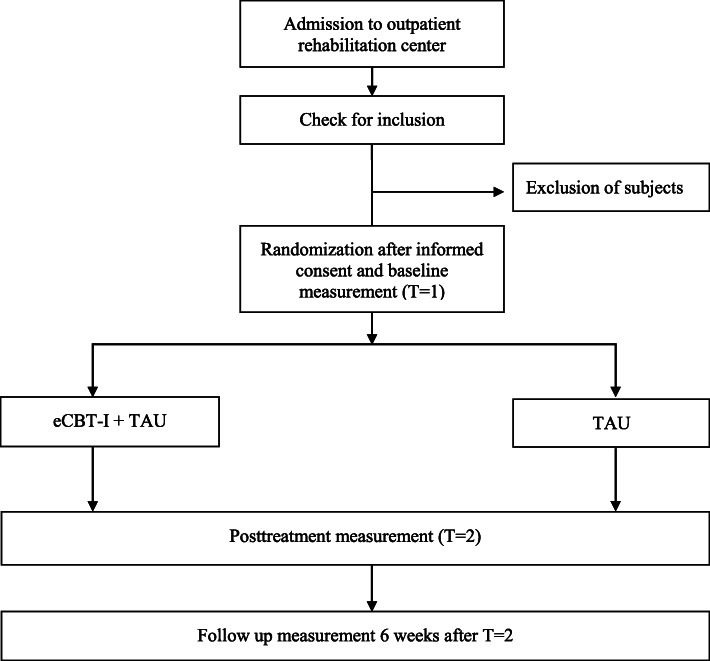
Flowchart of study. eCBT-I, online cognitive behavioral therapy for insomnia; TAU, treatment as usual

The randomized controlled trial (RCT) uses a multicenter, prospective, randomized, open-label, blinded end point study design (PROBE) [19] to compare eCBT-I with TAU. A total of 48 patients will be randomly assigned to the eCBT-I or TAU group. Assessment will be performed at baseline (T1), 1 week after the 6-week intervention period (T2), and after 6-week follow-up (T3). The total duration of participation is 14 weeks. See Fig. 1 for a flowchart.

### Recruitment, randomization, blinding, and treatment allocation

Participants will be recruited from five outpatient rehabilitation centers spread over the Netherlands (Heliomare Rehabilitation at Wijk aan Zee, Reade at Amsterdam, Rehabilitation Friesland at Beetsterzwaag, Basalt Rehabilitation at Delft and The Hague, and Adelante at Hoensbroek). Patients that are eligible based on the inclusion and exclusion criteria will be asked to participate. After informed consent is given, participants will complete the baseline measurement. Participants will then be randomly assigned to the eCBT-I or the TAU group. All participants will continue with standard rehabilitation care for various complaints, which do not specifically address insomnia (see Table 2). As complaints differ between persons, interventions differ as well. This care can include psychotherapy aimed at mood or other psychopathology, therapy aimed at cognitive functioning, physiotherapy, fitness, occupational therapy, and social work. The therapy dosage depends on different needs and capacity of participants. The variation in rehabilitation care between participants is expected to be similar for both groups and will be registered at follow-up by their clinicians. The TAU group will only receive standard rehabilitation care and can receive the eCBT-I after the study period. Participants can leave the study at any time for any reason without consequences. In case of withdrawal, participants will be asked to still do the measurements to control for attrition bias and a new patient will be included in accordance with the procedure. The measurements and intervention are not likely to have adverse consequences. Randomization will be done by a research randomizer program (www.randomizer.org) using permuted blocks to balance participants equal to both groups within centers. Treatment assignment is blinded, as researcher assistants, clinicians, and participants are unaware of number of included participants per center and not informed of this randomization procedure. Treatment allocation is not blinded for therapist and participant, as the nature of cognitive behavioral therapy does not permit blinding, but will be concealed until participants are recruited and baseline measurements are finished. Outcome assessment is self-reported and therefore not blinded for participant, but potential assessment bias of clinician or data collector on outcomes is excluded. Data collector is blinded for treatment allocation as well. Research assistant responsible for data input is blinded. See Table 3 for an overview of all measurements.

**Table 2:** Overview of treatment as usual (TAU)

TAU is standard rehabilitation care for various complaints, which do not specifically address insomnia. As complaints differ between persons, interventions differ as well.^a^ This care can include:	
	­ Neuropsychological assessment
	­ Psychotherapy aimed at mood or other psychopathology (online and/or face-to-face)
	­ Cognitive therapy
	­ Physiotherapy
	­ Fitness
	­ Occupational therapy
	­ Social work
	­ Vocational therapy

^a^Both groups will receive treatment as usual; dosage depends on their individual needs and capacity of participants. The variation in rehabilitation care between participants is expected to be similar for both groups and will be registered at follow up by their clinicians

**Table 3:** Assessment measures and time-points

	Enrolment	Baseline T1	Posttreatment T2	Follow-up T3
Eligibility screening	X			
Stop bang questionnaire	X			
Informed consent	X			
ISI		X	X	X
PSQI		X	X	X
Sleep diary		X	X	X
DMFS		X	X	X
HADS		X	X	X
CFQ		X	X	X
USER-P		X	X	X
Questionnaire received treatment				X

Questionnaire “received treatment” collects information on given standard rehabilitation care (TAU) during study period, reported by their clinicians

*ISI* Insomnia Severity Index, *PSQI* Pittsburg Sleep Quality Index, *DMFS* Dutch Multifactor Fatigue Scale, *HADS* Hospital Anxiety and Depression Scale, *CFQ* Cognitive Failure Questionnaire; *USER-P* Utrecht Scale for Evaluation of Rehabilitation—Participation, *T1* week 1, *T2* week 7, *T3* week 14

### Eligibility criteria

Participants are eligible for inclusion if diagnosed with stroke or TBI and insomnia disorder according to DSM-5 criteria. Furthermore, they should be aged 18 or older and capable of using the Internet. Exclusion criteria are untreated sleep apnea, current or expected treatment with a main focus on fatigue or sleep during the study, unstable medication regiments, use of medication with insomnia as side effect, alcohol or drug abuse, and a major untreated or unstable medical or psychiatric condition. Users of sleep medication will be encouraged to finish medication before enrolment or to keep intake stable during the study period. Depending on type and dosage of medication, the physician will determine the period needed to exclude the influence of finishing sleep-related medication on the outcome measures.

### Intervention

The eCBT-I comprises six guided weekly sessions provided online, combined with two face-to-face sessions of 60 min and a smartphone diary-app for daily reporting of sleep times and subjective sleep quality. Each session is structured around one topic and contains specific information, assignments and testimonials of two patients with insomnia after brain injury to illustrate sleep problems and homework assignments. All participants will receive online personal feedback after each session and will be encouraged to practice daily with the provided exercises, downloadable within the eHealth intervention on a daily basis. Participants can start with the next session after they have read their personal feedback. They can contact their therapist at any time by means of the integrated email function. Participants will be encouraged to complete the diary every day by their therapist.

The eCBT-I is based on well-established CBT-I components and includes behavioral and cognitive techniques. These techniques contain sleep hygiene education, stimulus control, sleep restriction, cognitive restructuring, activation, relaxation, and fatigue and stress management. The eCBT-I has been adjusted to people with acquired brain injury in general, both with respect to content and the way of conveying information. Content adjustments include specific education about the nature and treatment of insomnia after acquired brain injury, testimonials of patients with insomnia following acquired brain injury, and adaptations to cognitive impairments due to brain damage. Information is given in clear and short texts and is visually supported. An option is included to allow for listening to an audio version of the texts. All sessions follow the same structure, with repetition of key points. Specific feedback suggestions for each session will be provided for the therapists.

The length of our eCBT-I protocol of 6 weeks is comparable with eCBT-I protocols in other studies. The CBT-I protocol in other studies examining efficacy in ABI consisted of 4 to 8 weekly sessions [12–17], and 6 weeks for eCBT-I [18]. In a review on the efficacy of eCBTI in the general population, an average length of 5.5 weeks was found, with a range of 2–9 weeks [11].

The eCBT-I will be given by an experienced registered healthcare psychologist, trained in using the eHealth intervention. Adherence will be monitored by checking frequency of registration in the sleep diary app, time spent online, and online assignments done. For a detailed description of the intervention per week, see Table 1.

### Outcomes

#### Sleep outcome measures

The primary outcome measure is the change in insomnia severity measured with the Dutch version of the Insomnia Severity Index (ISI) at posttreatment. The ISI consists of 7 items and uses a 5-point scale to measure to which extent participants experience insomnia. The total score ranges from 0 (no insomnia) to 28 (severe insomnia). A cut-off of 10 is used to indicate clinical levels of insomnia in this study, similar to other studies [20–22]. The Minimally Clinical Improvement Difference (MCID), which indicates the minimal improvement to be clinically significant, is a reduction of six points [23]. The internal consistency is adequate (Cronbach’s alpha = 0.74–0.78). The ISI is selected as it is sensitive to treatment response [22, 24] and used in comparable research worldwide, including the Netherlands [20, 21]. Secondary sleep outcome measures include overall sleep disturbances assessed with the Pittsburgh Sleep Quality Index [25] and the following sleep features derived from the sleep diary app: total sleep time, sleep onset latency, number of nocturnal awakenings, sleep efficiency, and subjective sleep quality.

#### Other outcome measures

Secondary outcome measures cover fatigue, anxiety and depression, subjective cognitive functioning, and societal participation. Fatigue after acquired brain injury will be assessed with the Dutch Multifactor Fatigue Scale (DMFS). The DMFS measures 5 aspects of fatigue: impact of fatigue, mental fatigue, signs and direct consequences of fatigue, physical fatigue, and coping with fatigue. All subscales of the DMFS showed sufficient to good reliability (Cronbach’s alpha = 0.70 to 0.91), good convergent validity with an existing fatigue scale, and good divergent validity with measures of mood and self-esteem [26]. Anxiety and depression symptoms will be assessed with the Dutch version of the 14-item Hospital Anxiety and Depression Scale (HADS). The reliability of the HADS is good (Cronbach’s Alpha = 0.71 to 0.90) as is the test-retest reliability (0.86–0.90) [27]. Cognitive Failure Questionnaire (CFQ) is a measure of subjective cognitive functioning. Internal consistency is good (Cronbach’s alpha = 0.88) as is the test-retest reliability (0.83) [28]. The Utrecht Scale for Evaluation of Rehabilitation—Participation (USER-Participation) is a questionnaire to rate objective and subjective participation after rehabilitation. Internal consistency is satisfactory (Cronbach’s Alpha = 0.70–0.91), and test-retest reliability is 0.65 for the frequency scale, 0.85 for the restrictions scale, and 0.84 for the satisfaction scale [29, 30].

### Other study parameters

Demographical, injury-related, and clinical variables which may influence the treatment effect will be registered: age, gender, diagnosis, time since injury, insomnia duration, use of prescribed sleep medication, use of other medication, comorbid psychiatric and somatic disorders, educational level, and currently being employed. Possible presence of sleep apnea will be screened with the Stop-Bang questionnaire [31] in participants that have not been evaluated for its presence with the gold standard overnight polysomnography [32].

### Sample size calculation

Calculation of power and group size in a repeated measures design requires an estimate of the expected intraclass correlation coefficient (ICC, within subject correlation). We therefore resorted to a recent randomized trial of Dekker and colleagues (2020) on the effect of eCBT-I on ISI in people with insomnia. We used this intraclass correlation coefficient as indication of follow-up of ISI assessments across 6 weeks in our study population with acquired brain injury. That study reported an ICC of 0.54 for ISI assessments repeated across 6 weeks in 175 people suffering from insomnia without brain injury [33]. Calculation of power and group size moreover requires an estimate of the expected treatment effect size. Whereas treatment effects of eCBT-I are often reported to be of moderate to large size, we preferred to be somewhat conservative and expect a somewhat smaller than moderate effect in the population of TBI and stroke and therefore used *f* = 0.20. Calculation of the required sample size using G*Power [34] for ANOVA, with two repeated measures and ICC of 0.54, indicated that 48 completers would provide (2 × 24), at a significance of alpha = 0.05, sufficient power (1-beta = 0.80) for a minimal detectable time-by-group interaction effect of *f* = 0.20 (small to moderate).

### Statistical analyses

Data analysis will be performed with SPSS 23 (IBM; Armonck, USA). Means and standard deviations of the demographic and injury-related variables and the clinical characteristics collected at baseline will be calculated. As normality assumptions may not be met with this small sample size, data distributions and all model assumptions will be checked for all analyses. If model assumptions are not met, data will either be transformed or analyzed using a non-parametric test, as appropriate. For data not normally distributed, median and interquartile range will be reported. Independent *T* tests will be used to check for an imbalance between groups. Nominal variables will be checked with *χ*^2^ tests and ordinal variable with the Mann-Whitney Test. Statistical significance will be set at a *p* value of 0.05. To accommodate likely occasional missing days, the repeated measures obtained by diaries will be analyzed with mixed effect models. The treatment effects will be examined using a repeated-measures ANOVA. In the case of significant baseline imbalance, post hoc analysis of covariance (ANCOVA) will be used on the change scores of the outcome measures with baseline scores as covariates. Intention-to-treat analyses will be conducted and presented as primary results. Additionally, a per-protocol analysis will be performed. Also, we will compare the eCBT-I and TAU group with regard to the percentage of participants who have improved (reduction ≥ 6 points on the ISI) and recovered (ISI < 10). We will use mixed effect regression models to explore whether treatment efficacy, i.e., the time-by-treatment effect, is modified by time since brain injury and by diagnosis (stroke versus TBI).

## Discussion

To date, there are three randomized controlled trials with small samples evaluating the effects of CBT-I for people with acquired brain injury [12, 14, 18]. Taken together, number of treated participants in the CBT-I studies was 34 patients with a stroke or TBI. Considering this scarcity, more research is needed as we concluded in our recent review on this topic [35]. This study is designed primarily to evaluate the effect of eCBT-I following stroke and traumatic brain injury and therefore expected to provide insight on effects on sleep, fatigue, subjective cognitive functioning, emotional well-being, and societal participation. It uses an online intervention that can be disseminated at low cost to professionals when found effective. Also, this study will explore who will benefit from treatment. Strength of this study is the assessment of ID according to DSM-5 criteria; the inclusion of information on severity of brain injury and common comorbid factors, such as sleep apnea; and the use of secondary outcome measures for cognitive functioning, emotional wellbeing, fatigue, and societal participation. As a result, this study is expected to strengthen the body of evidence of effectiveness of CBT-I for people with acquired brain injury and provide clinicians with evidence to help formulate future guidelines for the treatment of insomnia following stroke and TBI.

There are several limitations to this study that should be noted. In contrast to earlier research, we will combine stroke and TBI patients in our study, as insomnia complaints are comparable for those diagnoses. Moreover, other symptoms like cognitive and emotional consequences of the brain injury are similar as well. Our hypothesis is that eCBT-I will be similar effective for stroke and TBI patients. Combining both diagnoses may increase inclusion rate and therefore shorten study period. If effective, the eCBT-I will be earlier accessible in time for those who need it. A potential limitation of this study is that differential effects between types of brain injury will be missed. This will be explored with mixed effect regression models. A second potential limitation is that we asses sleep only by subjective sleep measurements, as insomnia symptoms are our primary focus of interest. It would be interesting to use objective measures of sleep as is recommended as a standard research assessment of insomnia [36]. However, we chose not to do this, as we expected this to be an additional burden on the participant that could negatively impact treatment adherence and increase drop-out. At last, participants in both groups will have the benefits of personal attention and standard rehabilitation care tailored to their needs. However, placebo and nocebo effects cannot be completely excluded, as participants are aware of treatment assignment and expectation may influence outcome.

## Trial status

Protocol version 2, September 19, 2017. Recruitment started in January 2018 and is expected to be completed in September 2020. The final participants are expected to complete their assessments at the end of 2020.

## Data Availability

The datasets used and/or analyzed during the current study are available from the corresponding author on reasonable request.

## Abbreviations

ABI: Acquired brain injury
CBT-I: Cognitive behavioral therapy for insomnia
eCBT-I: Online cognitive behavioral therapy for insomnia
ID: Insomnia disorder
ISI: Insomnia Severity Index
TAU: Treatment as usual
TBI: Traumatic brain injury
